# Caterpillars suppress nocifensive behaviours during the quiescent ‘sphinx’ state

**DOI:** 10.1098/rsbl.2025.0124

**Published:** 2025-08-20

**Authors:** Gayathri Kondakath, Isabel M. Messinger, Annushka Veliko-Shapko, Barry Trimmer

**Affiliations:** ^1^ Department of Biology, Tufts University, Medford, MA, USA

**Keywords:** nociceptive modulation, pain, *Manduca sexta*, insect larvae, defensive posture, nociceptive tolerance

## Abstract

Noxious (harmful) stimuli are detected by a specialized sensory process known as nociception, which typically evokes defensive behaviours to minimize tissue damage. While sensitization of nocifensive responses by intense or repetitive stimuli is well-documented in insects, instances of deliberate nociceptive suppression are relatively unexplored, particularly within natural behavioural contexts. Here, we describe a behavioural state in the tobacco hornworm (*Manduca sexta*), termed the ‘sphinx’ state, characterized by a distinct ventrally curled posture of the head and thorax following gentle disturbance. One of the striking characteristics of this state is that the larvae exhibit reduced responsiveness to noxious stimuli, indicating nociceptive downregulation. We also observe an overall reduced behavioural responsiveness to innocuous stimuli. Our surgical experiments show that the cerebral ganglion is essential to initiate the sphinx state. Overall, this discovery reveals a novel instance of active behavioural modulation in insects and highlights the flexibility of nociceptive responses, challenging the notion of nociception as strictly hard-wired and stereotyped.

## 1. Introduction

Most animals are under strong selection to survive threats, including predation, parasitism and pathogens, leading to the evolution of several defensive phenotypes. Larval lepidopterans are slow and extremely vulnerable to predation during the most important activity of their life stage, feeding [1]. Chemicals act as a primary mode of defence in these animals, via allelochemical sequestration and immune responses that encapsulate parasitoid larvae and other pathogens. In addition, the caterpillars employ various morphological and behavioural adaptations against these threats [2]. Using integumental structures such as spines and hairs, larvae can strongly adhere to leaf surfaces and protect their exposed appendages from potential attacks. Coloration is also used protectively by signalling unpalatability via bright warning pigmentation or by cryptic patterning to match their substrate and inedible objects. Finally, caterpillars can mimic organisms that are dangerous to their predators [3].

Instances of static crypsis and mimicry are likely to be successful defensive strategies only if normal movements are suppressed. For example, thanatosis or ‘death feigning’, a common behavioural state to escape predation, is accompanied by significant drops in heart rate, respiratory rate and body temperature in the American opossum [4], while drastically diminished abdominal ventilatory movements are observed in several insects [5]. In addition to the suppression of baseline physiological activity, tolerating noxious stimuli would be critical to maintain the facade of thanatosis, as nociceptive responses are often vigorous and rapid in nature.

*Manduca sexta* exhibits a well-defined and adaptable nociceptive system, making it a valuable model for studying how organisms detect, respond to and modulate noxious stimuli. *Manduca* larvae exhibit an extensive repertoire of nocifensive behaviours including withdrawal, strike, cocking, thrashing and quivering [6]. These responses vary depending on the stimulus location along the anteroposterior axis of its body [7]. Stimulating the head or thorax with a noxious stimulus causes ‘withdrawal’ away from the stimulus. Conversely, the larvae rapidly ‘strike’ with their head when the posterior segments are stimulated.

Our study identifies and characterizes a behavioural state marked by a distinctive posture in *M. sexta*. Termed ‘sphinx’ state, this behaviour represents a rare instance of deliberate and reversible suppression of nociceptive responses in insects. We specifically investigated three questions: (i) how do we reliably induce sphinx state, (ii) is the brain essential for transition into sphinx state, and (iii) how is behavioural responsiveness to both noxious and innocuous stimuli affected during sphinx state? We examined these questions using surgical and behavioural experiments. We first evaluated the tendency of larvae to assume the sphinx posture in response to various mechanical stimuli. Next, to assess the role of central neural input, we surgically transected the nerve connectives to the head ganglia, which showed that brain connectivity and thus descending neural input are essential for initiating the sphinx state. Finally, we examined behavioural modulation during the sphinx state by measuring defensive responses to noxious stimuli as well as proleg withdrawal reflexes (PWR) to planta hair touch. Notably, during the sphinx state, larvae exhibited a selective and reversible suppression of nociceptive responses, along with partial suppression of responsiveness to innocuous touch on the planta hair. Although this behavioural state has been briefly noted in previous studies, it has not been systematically characterized to date. These findings reveal a previously unrecognized form of sensory modulation in insects and suggest that invertebrate nervous systems can exert strong state-dependent control over defensive responsiveness.

## 2. Results

### (a) ‘Sphinx’ state in caterpillars

**Figure 1.**
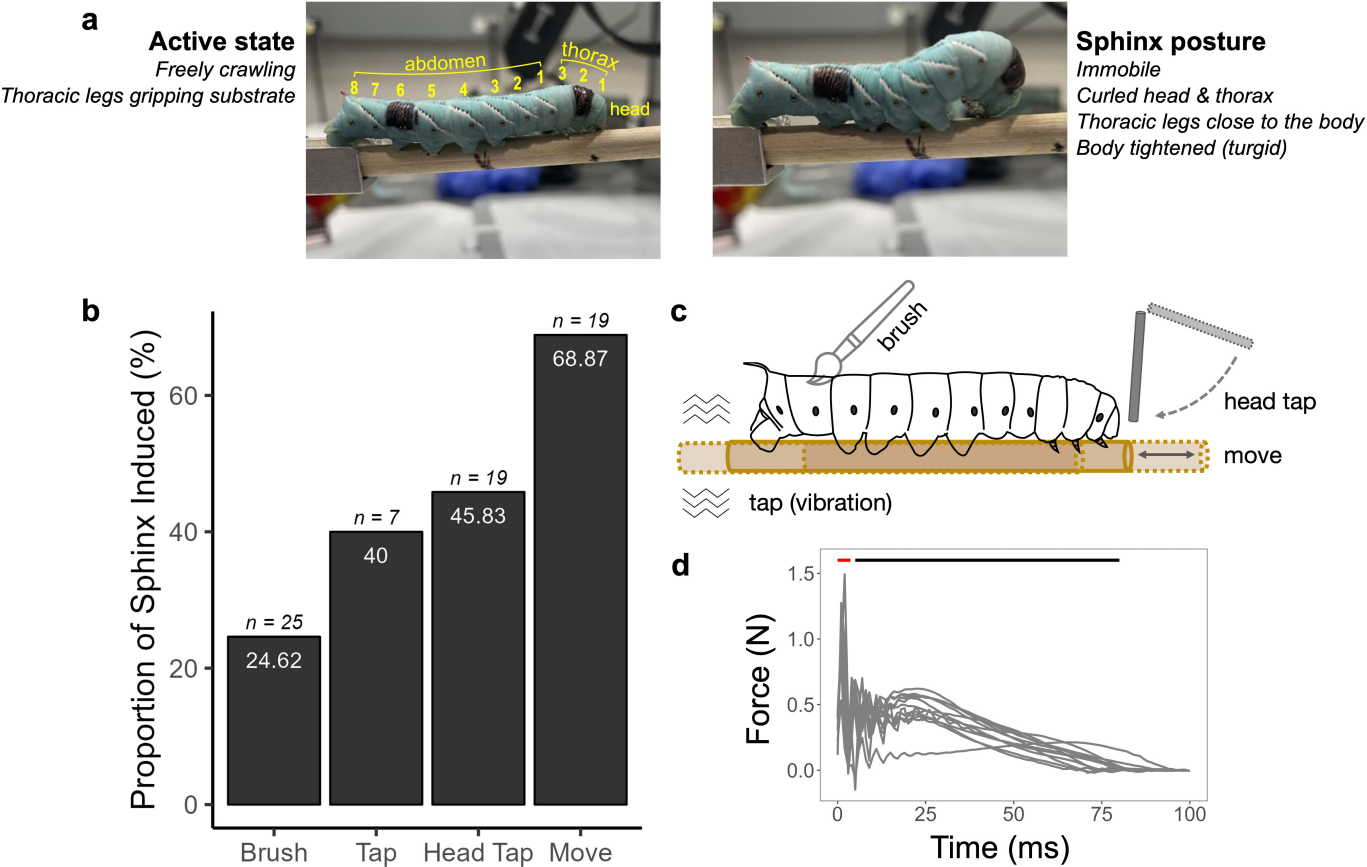
Quiescent ‘sphinx’ state is induced by mechanical stimuli in *Manduca sexta.* (a) ‘Sphinx’ posture (right) features ventral curling of the head and thorax, unlike the active posture (left, with body plan). Black paint spots on the sixth abdominal segment (A6) and the second thoracic segment (T6) mark areas for thermal stimulation. (b) Sphinx state is induced by various mechanical stimuli. (c) Description of the stimulus methods. (d) Overlaid head-tap force traces aligned to stimulus onset. Red bar marks the fast, transient phase; black bar marks the slower, sustained phase.

Upon tactile disturbance, *M. sexta* larva adopts a motionless stance with thoracic legs held tightly against the body and the head drawn underneath (figure 1a), giving the impression of swollen anterior segments (henceforth, the ‘sphinx’ posture). The caterpillar’s body during this state is more turgid than during active crawling or resting.

### (b) Sphinx state is induced by mechanical stimulation/disturbance

The sphinx state can be reliably triggered through various mechanical disturbances (figure 1c). Interestingly, the sphinx state was most frequently triggered by translational movement of the substrate (figure 1b). However, as the head-tap stimulus kept the animal in a fixed position and was highly reproducible, it was used for the subsequent experiments. The tap force consistently shows a fast, transient component (approx. 5 ms long) followed by a slower, longer lasting component of the stimulus (figure 1d).

### (c) *Manduca sexta* does not enter sphinx state spontaneously

To determine whether the sphinx state is an ‘evoked’ response or a ‘spontaneous’ one, we monitored instances of sphinx states in caterpillars that were left undisturbed for an extended period. Our observations indicated that when they were separated from external mechanosensory stimuli for 24 h, none of the 20 larvae entered the sphinx state; instead, they were only observed engaging in crawling, feeding and defaecating. This suggests that the sphinx state is an ‘evoked’ response that is initiated on sensing an external stimulus.

### (d) The brain is essential to enter the sphinx state

**Figure 2.**
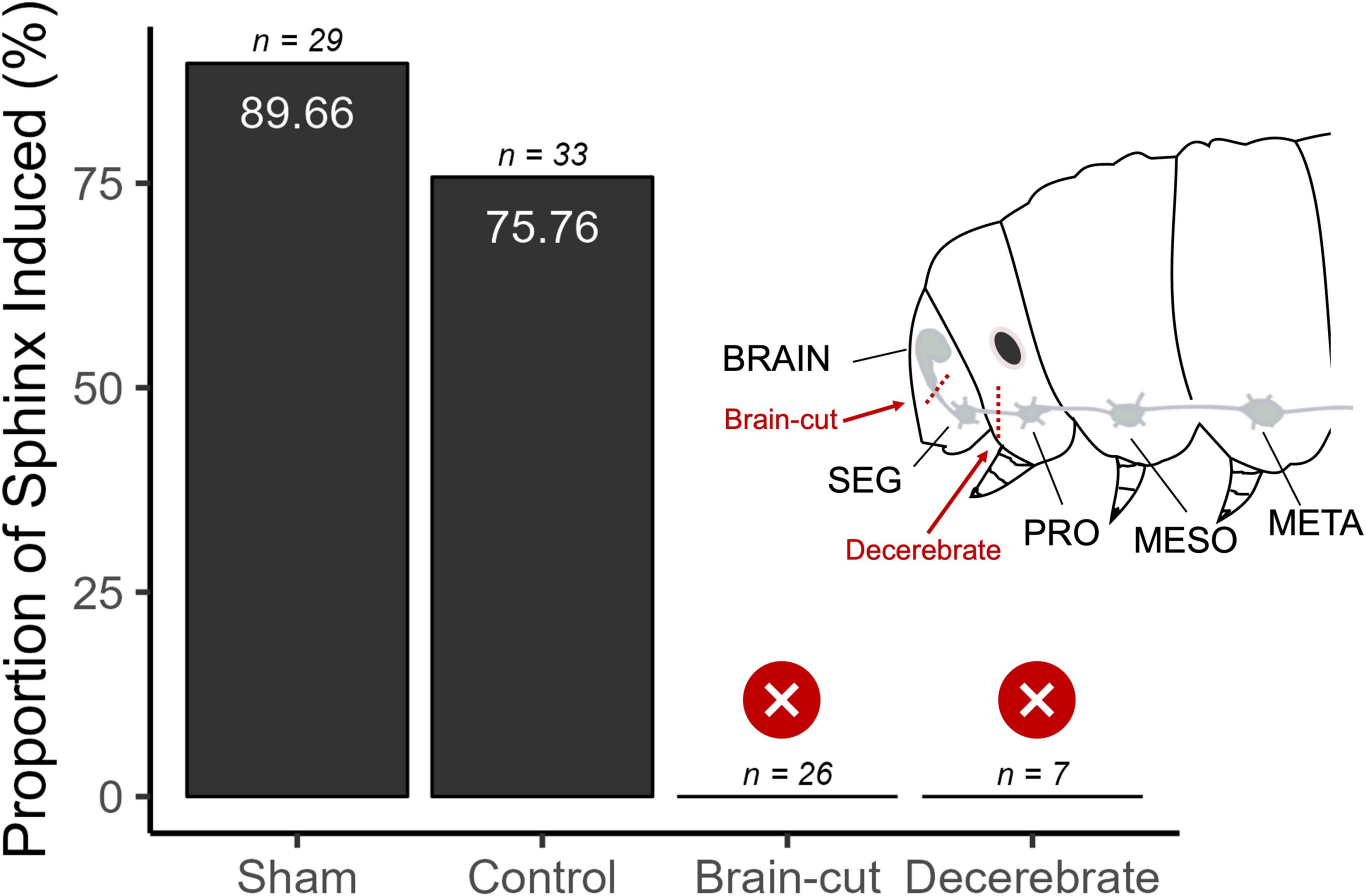
Brain connectivity is essential for initiating the sphinx state. Decerebrate (connection between SEG and prothoracic ganglion cut) and brain-cut (connection between brain and SEG cut) larvae do not enter the sphinx state. SEG: suboesophageal ganglion.

To investigate the role of descending control of the sphinx state, we surgically severed the connection between the suboesophageal ganglion (SEG) and the prothoracic ganglion (‘decerebrate’) and observed its effect on the likelihood of sphinx state induction. These observations were compared to sham (surgical protocol minus severing) and control (intact animal) groups. To further examine the role of the brain, we introduced another treatment group where we severed the neural connection between the brain and the SEG (‘brain-cut’). Both the brain-cut and decerebrate groups were significantly less likely to enter the sphinx state relative to the control group (brain-cut: *z* = −3.36, *p* = 0.0008; decerebrate: *z* = −2.36, *p* = 0.018, Firth’s logistic regression, figure 2). The sham and control groups did not show a significant difference (*z* = 1.31, *p* = 0.189, Firth’s logistic regression), indicating that the brain is essential for the sphinx state and the SEG alone is not sufficient to initiate it.

### (e) Caterpillars are less responsive to noxious stimuli in the sphinx state

**Figure 3.**
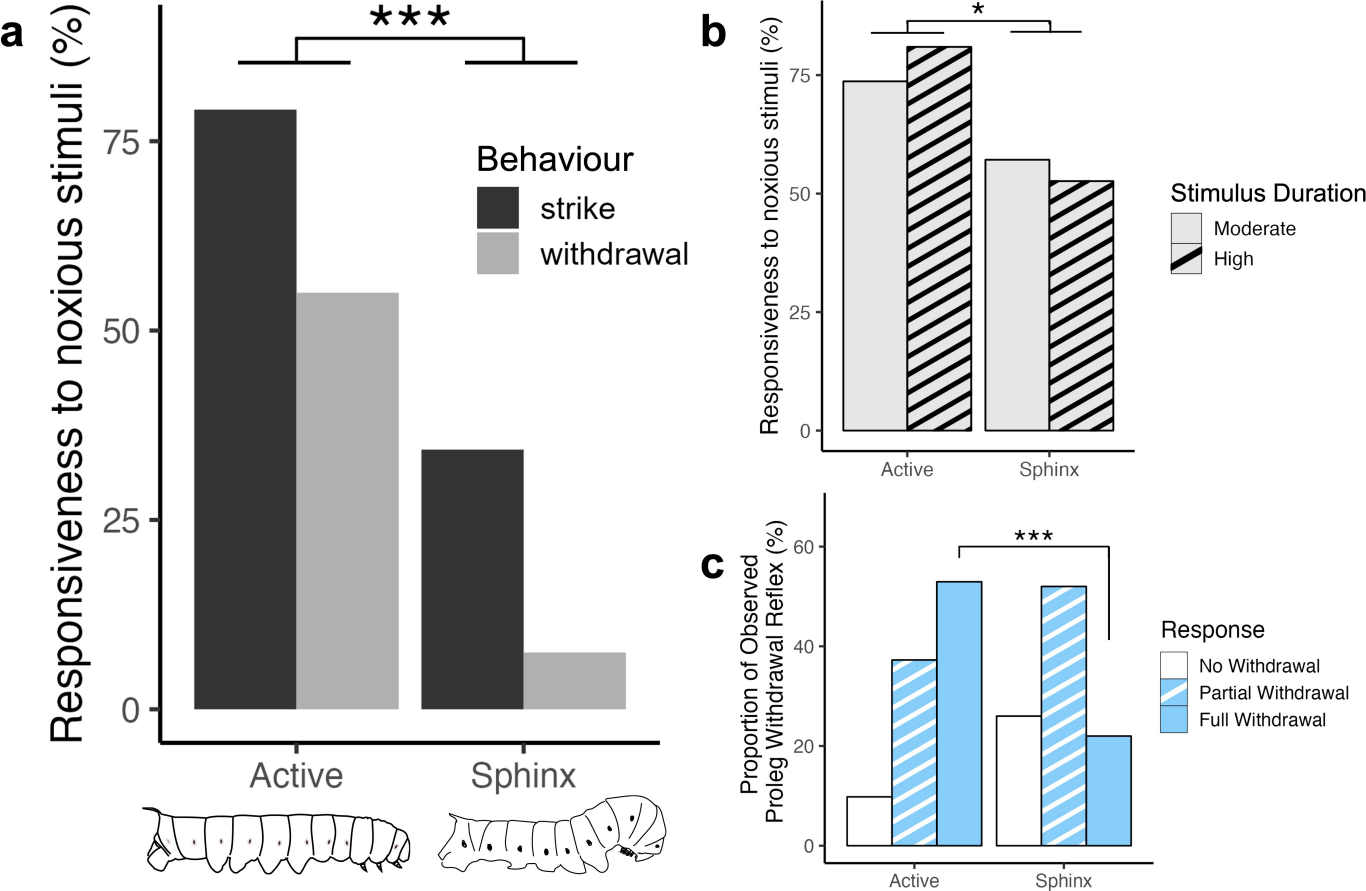
Caterpillars are less behaviourally responsive to noxious and innocuous stimuli in the sphinx state. (a) Caterpillars in sphinx state have significantly fewer nociceptive responses (*p* = 0.009, binomial GLMM, *n* = 11 larvae, 60 trials for T2, 59 trials for A6). (b) Significant nociceptive suppression continues at moderate (400 ms) and high (500 ms) stimulus duration (*p* = 0.037, binomial GLMM, *n* = 20 larvae, 80 trials). (c) Full proleg withdrawal reflex in response to planta hair stroke is significantly reduced in the sphinx state (*p* = 0.008 (Bonferroni-corrected), *n* = 10 larvae, 101 trials).

*Manduca* larvae in the sphinx state were significantly less likely to respond to a noxious stimulus (*z* = 2.57, *p* = 0.009, binomial generalized linear mixed model (GLMM), figure 3a, *n* = 11 larvae, 119 trials). Since A6 (sixth abdominal segment) and T2 (second thoracic segment) stimulation reliably trigger the distinct behaviours of strike and withdrawal, respectively [7], we were able to assess how the sphinx state affects these behaviours. There was no interaction between the stimulated site (T2 or A6) and the behavioural state, indicating that strike and withdrawal were not differentially affected by the sphinx state (*z* = 1.643, *p* = 0.100, binomial GLMM). As the sphinx state significantly suppressed overall nociceptive responsiveness, and there was no differential effect on strike versus withdrawal, we conclude that both behaviours were significantly reduced in the sphinx state. We further tested whether sphinx-mediated nociceptive suppression persisted under longer stimulation durations by assessing responses at 400 and 500 ms in the same animals across both behavioural states. There was a significant main effect of behavioural state (*z* = 2.088, *p* = 0.037, binomial GLMM, figure 3b, *n* = 20 larvae, 80 trials), indicating that suppression of nocifensive responses during the sphinx state remained robust at longer durations. No significant interaction was observed between behavioural state and stimulus duration (*z* = 0.624, *p* = 0.53, binomial GLMM), suggesting that the effect of the sphinx state was consistent across both durations. There was no significant difference in responses between the 400 and 500 ms stimulus durations, indicating that the stimulus duration within this tested range does not strongly modulate behavioural outcomes.

### (f) Caterpillars in the sphinx state have reduced responsiveness to the proleg withdrawal reflex

The PWR is a reliable and well-studied reflex with a well-characterized underlying neural circuit [8]. At rest, the prolegs of the *Manduca* larva are extended. Responses to planta hair stimulation evoke a ‘partial withdrawal’ when the crochets are disengaged, and ‘full withdrawal’ when the proleg is retracted completely away from the substrate [8]. Larvae in the sphinx state have a significantly reduced proleg withdrawal response (*χ^2^* = 11.373, *p* = 0.003, Pearson’s χ^2^-test, figure 3c, *n* = 10 larvae, 101 trials). Furthermore, analyses revealed some interesting patterns. First, larvae in the sphinx state exhibit significantly lower ‘full withdrawal’ reflex compared with when they are active (*p* = 0.008, figure 3c). Second, the proportion of ‘no withdrawal’ is not significantly different across active and sphinx states, indicating that the animals do not show differences in sensitivity to stimuli (*p* = 0.20).

## 3. Discussion

Our study identifies and describes the ‘sphinx’ state, frequently observed in many lepidopteran larvae. Noted in past studies as a resting posture [6,9], our study is the first to characterize it as a behavioural state with associated physiological modulations. We show that this is a reversible, quiescent state that caterpillars assume when they experience mechanical disturbance in their surroundings.

We demonstrate an interesting and unexpected physiological feature of the sphinx state—nociceptive suppression. Nociceptive sensitization, in the form of hyperalgesia or allodynia, has been demonstrated and studied in *Drosophila melanogaster* [10–13] and *M. sexta* [6,9,14]. Previous work has shown that *Manduca* exhibits both peripheral and central sensitization in response to thermally [15] and mechanically noxious stimuli [6,14]. Here, we report a novel instance of natural nociceptive downregulation in caterpillars. Suppression of nociceptive response has also been demonstrated in *Manduca* larvae during a quiescent developmental phase named ‘moult-sleep’ [16] and during emergence of the parasitoid wasp, *Cotesia congregata*, through the larval body wall [17]. It would be valuable to further study the mechanism that governs the suppression of nociception during the sphinx state. An interesting possibility is the state-dependent uncoupling of sensory and motor circuits, as seen in *Drosophila*, where a looming stimulus that triggers an escape response in perching flies also evokes landing responses when they are flying [18]. In *Manduca*, the sphinx-inducing signal before noxious stimuli may play a similar role in decoupling the nociceptive signals and vigorous motor responses like strike or withdrawal. Alternatively, the significantly reduced nociceptive responsiveness is also reminiscent of pre-pulse inhibition, wherein startle responses to sudden, unexpected stimuli are diminished when preceded by a weak stimulus of another modality. This effect has been extensively studied in the marine mollusc *Tritonia diomedea*, where a vibrational stimulus (pre-pulse) delivered before an aversive skin stimulus suppresses the escape swim motor programme [19]. This suppression is mediated through pre-pulse-induced presynaptic inhibition of afferent neurons transmitting the startle signal, as well as post-synaptic inhibition acting on multiple downstream sites within the swim circuit [20]. There may exist such an inhibitory network in *Manduca* that comes into play when the sphinx state is triggered by gentle disturbances in the environment.

We also show that the response to an innocuous stimulus, such as a planta hair stimulation, is reduced during the sphinx state. The partial proleg retraction is driven by the principal planta retractor muscle (PPRM), with its motoneuron (PPR) making a strong, monosynaptic connection with the planta hair sensory neurons. Conversely, the full proleg retraction requires recruitment of the accessory planta retractor muscle (APRM), and the connections to the APR motoneurons are weaker [8,21]. Our experiments indicate that in the sphinx state, there are fewer instances of full withdrawals, while partial withdrawals persist, likely because sphinx-mediated inhibition primarily suppresses APR motor neuron recruitment. PPR motor neuron has a lower spike threshold than APR such that deflection of a single hair can elicit firing in PPR [21,22]. This difference in the strength of sensory input to the different motoneurons could explain why full withdrawal, but not partial withdrawal, is suppressed during the sphinx state. It is also possible that inhibitory interneurons in the polysynaptic circuits preferentially limit APR activity. Another potential mechanism could be the direct suppression of peripheral sensory neurons, as seen in crayfish, where sensory afferents of a stretch receptor display primary afferent depolarizations (PADs) [23,24]. Future intracellular recordings from the motor and sensory neurons during the sphinx state would allow us to identify the mechanism of inhibition.

Our surgical manipulation experiments demonstrated that the SEG alone is insufficient to induce the sphinx state in the absence of brain connectivity—an intriguing observation, as caterpillars with severed brain connections continue to exhibit normal locomotory behaviours such as crawling [25]. This result suggests that the absence of sphinx state after surgery is not attributable to a lack of muscle recruitment, but that a descending command from the brain is necessary to initiate the posture. Mechanosensory neurons from the head and its sensory appendages project to the SEG, with some extending to the first thoracic ganglion [26]. If the effect on the sphinx state was due to sensory impairment from the surgery, it would not have been entirely abolished in the brain-cut group. Therefore, it is unlikely that this effect was due to sensory disruption alone. The most likely candidate for exerting such control is the central complex, a multisensory neuropil that processes a variety of visual, mechanosensory and olfactory signals [27]. In *Drosophila*, it is known to exert dopaminergic control on the states of arousal, including stress-induced arousal, wakefulness, ethanol-induced hyperactivity and aggression [27].

Our results add to the growing body of work which suggests that insect nociception is subject to descending modulation [28]. Previous studies have demonstrated the role of neuropeptidergic systems in modulating nociception in *Drosophila* [12,29–31]. Food-motivated bumblebees have been observed to tolerate noxiously heated feeders to access high-sucrose food sources [32]. Fruit flies display starvation-induced reduction in nocifensive behaviour, regulated by signalling of leucokinin, another neuropeptide [33]. Interestingly, *Manduca* also shows a similar upregulation of leucokinin in the central nervous system during starvation [34]. However, this study did not investigate the nociceptive effects associated with the leucokinin upregulation. Another descending GABAergic pathway suppresses noxious inputs via activation of glucose-sensing neurons in starved fly larvae [35]. This would enable insects to prioritize foraging and disregard noxious stimuli when they experience a nutrient-depleted state.

The day-long undisturbed monitoring experiments suggest that the sphinx state is not merely a resting posture; it may serve a defensive function, and the animals do not appear to enter this behavioural state spontaneously. Anecdotally, caterpillars in the wild are often described (and photographed) in the sphinx state, but we believe they may be responding to the observer’s presence or another mechanical disturbance. It resembles the ‘freezing’ behaviour exhibited by several animals upon detection of imminent danger, which is quite common in large caterpillars and not limited to sphingid moth larvae. Such defensive postures could confer protection from predation by insectivorous birds in the wild, thereby increasing the chances of survival to adulthood and subsequent reproduction [36]. This is achieved by appearing larger in size and, in species that have prominent ‘eyespot’ markings, it enhances the caterpillar’s overall resemblance to snakes [37]. However, it is also possible that the posture serves as camouflage or conveys other defensive signals to potential predators. The sphinx posture is reminiscent of tonic immobility (TI)—a behavioural state marked by stiff posture and pronounced tonic muscular activity. In crickets, the slow flexor motor neurons of the tibiae fire tonically to maintain the stiff posture, mimicking a dead conspecific [38]. The sphinx posture is probably maintained by tonic activity in the large intersegmental muscles of the thorax and anterior abdominal segments, which could impose a metabolic cost of this behaviour. Interestingly, during TI in stick insects, the threshold for nociceptive responses is raised [39], a pattern we also observed in the sphinx state. Nociceptive suppression potentially allows these organisms to effectively maintain the illusion that keeps predators at bay. This ability thus points to a significant evolutionary advantage in animals, highlighting the importance of adapting nociceptive processing based on environmental and behavioural contexts. Moreover, the state-dependent modulation of nociception in insects suggests that nociceptive circuits are more flexible and adaptable than previously believed.

Our experimental results provide an opportunity to develop *Manduca* as a tractable model for studying how intrinsic circuits regulate and balance the expression of different behaviours, e.g. how is nociception suppressed or enhanced to ensure that vigorous movements are expressed only when it is appropriate to do so? In some instances, these movements could attract predators or dislodge the caterpillar from its substrate. Electrophysiological and neuroanatomical studies on intact or semi-intact preparations of *M. sexta* could help identify the mechanisms that bias the expression of different behaviours in various contexts.

## 4. Material and methods

### (a) Colony

Second- or third-day fifth-instar larvae of *M. sexta* (both sexes) were used for all experiments. The caterpillars were reared on an artificial diet at 27°C in a light : dark cycle of 17 h : 7 h following a standardized protocol [40].

### (b) Spontaneous sphinx state in undisturbed caterpillars

A vertical stack of 10 clear plastic vials was made by glueing the walls of the vials. Nail files were used to provide a rough substrate for the caterpillars to walk inside the vials, which were inserted into the vials via slits in foam plugs. A small block of food was placed at the free end of the stick. In each trial, four animals were placed in the stack (one per vial), and the vials were kept horizontally inside the incubator. The incubator was set to maintain a constant temperature of 27°C, and the light cycle was regulated using a timer (17 L : 7 D). An infrared night vision camera (Wyze Cam V3 1080 p; Wyze Labs, Inc.) was used to record the behaviour for 24 h. Operational via a mobile app, the video was recorded using the timelapse function, which captured an image every minute for 24 h, since the sphinx state extends over longer timescales. The first hour after the placement in the incubator was excluded from analyses to allow for acclimatization to the new environment.

### (c) Testing responsiveness to stimuli

#### (i) Inducing sphinx state

Different types of mechanical stimuli tested for inducing sphinx state were: vibration (tapping on the substrate), mild mechanosensory stimuli (stroking the larval body with a paint brush), head tap (gently tapping the head of the caterpillar) and acceleration of the substrate (moving the dowel back and forth). The force of the head-tap stimulus was measured with a force sensor (MLTF500/ST, ADInstruments, Australia). A caveat of this method is the potential variability in tap intensity across experimenters. Although we standardized procedures through training with a force sensor, we lack the direct force measurements for every single trial. Therefore, while we assume relative consistency in the tap force, some variability cannot be ruled out.

#### (ii) Proleg withdrawal response

To induce the PWR, we used a paintbrush to gently touch the planta hair of a proleg. The experimental set-up consisted of two wooden sticks separated by a small gap (approx. 3 mm) to touch only the planta hairs of the unattached proleg, as holding onto a substrate tends to suppress the reflex [41]. The anterior prolegs of the caterpillar were placed on one stick, while the posterior prolegs were placed on the other, ensuring that the middle prolegs were suspended over the gap. This allowed for precise stimulation of the mechanosensory hairs located on the planta of the middle prolegs.

#### (iii) Nocifensive behaviours

We tested reactivity to noxious thermal stimuli [15,42] applied to the T2 (second thoracic segment) and A6 (sixth abdominal segment) body segments, separately. T2 stimulation and A6 stimulation reliably elicit a withdrawal and strike behaviour, respectively, in *M. sexta* [7]*.* The stimuli were delivered in a randomized order. The nocifensive responses are triggered using infrared (IR) lasers (Class 3B Laser; 808 nm, 400 mW) to produce highly localized and repeatable thermal stimuli. Previous studies have demonstrated that the nociceptive sensory neurons are activated by both mechanically and thermally noxious stimuli [42]. The caterpillar is placed approximately 30 cm from the laser, on a dowel attached to manipulators to control the target location on the body wall. Since the *Manduca* epidermis is infrared-reflective, a small patch of cuticle is coated with a thin layer of black paint (Rustoleum™, flat black, oil-modified alkyd) and the laser beam is focused on this point to create a localized heat pulse. We were able to reliably evoke nocifensive behaviours with a stimulus duration of 200 ms. For increased stimulus strength, we used 400 and 500 ms.

### (d) Surgery protocol

A specialized set-up was used to perfuse CO₂, keeping the caterpillar anaesthetized throughout the procedure. Positioned ventral side up, the nerve cord, including the SEG and prothoracic ganglia, was visible through the cuticle. The epidermis was incised, the nerve connective severed and the wound sealed with light-cure glue, ensuring the thoracic legs remained unbound. After surgery, the caterpillars recovered overnight before testing the next day. Sham surgeries replicated all steps except for cutting the connective. As the connectives are easily accessible without disrupting deeper tissue or muscle, it is unlikely that the procedure impaired the musculature required for sphinx behaviour.

### (e) Statistics

For the surgical manipulation experiments, we used Firth’s penalized likelihood logistic regression to account for complete separation in the data, where some treatment groups showed zero instances of the sphinx state. The treatment group was included as a fixed factor, and the binary outcome (sphinx state induced or not) was modelled using a logistic link function (S1).

For the nociceptive responsiveness experiments, we fit GLMMs with a binomial error distribution (and logit link function) (S2). Behavioural state (active/sphinx) and stimulation site (T2/A6) were fixed factors, and animal identity was used as a random factor. Response was coded as 1 if the animal performed ‘strike’ or ‘withdrawal’ and coded as 0 if there was no response. In addition, stimulus duration (400 or 500 ms) was included as a fixed factor in the analysis to assess its effect on behavioural responses during the sphinx state.

All data processing and statistical analyses were performed in R (v. 4.0.3 [43]). Data were visualized using the ‘ggplot2’ package v. 3.4.2 [44]. We used the ‘lme4’ package [45] for model fitting and the ‘emmeans’ [46] for pairwise *post hoc *comparisons.

## Data Availability

All data used to generate results are available from Figshare [47]. Supplementary material is available online [48].

## References

[1] Bernays EA. 1997 Feeding by lepidopteran larvae is dangerous. Ecol. Entomol. **22**, 121–123. (10.1046/j.1365-2311.1997.00042.x)

[2] Ruxton GD, Allen WL, Sherratt TN, Speed MP. 2019 Avoiding attack: the evolutionary ecology of crypsis, aposematism, and mimicry. Oxford University Press.

[3] Greeney H, Dyer L, Smilanich A. 2012 Feeding by lepidopteran larvae is dangerous: a review of caterpillars’ chemical, physiological, morphological, and behavioral defenses against natural enemies. Invertebrate Surviv. J. **9**, 7–34.

[4] Gabrielsen GW, Smith EN. 1985 Physiological responses associated with feigned death in the American opossum. Acta Physiol. Scand. **123**, 393–398. (10.1111/j.1748-1716.1985.tb07605.x) 3993399

[5] Nishino H, Sakai M. 1996 Behaviorally significant immobile state of so-called thanatosis in the cricket Gryllus bimaculatus DeGeer: its characterization, sensory mechanism and function. J. Comp. Physiol. A **179**, 613–624. (10.1007/BF00216126)

[6] Walters ET, Illich PA, Weeks JC, Lewin MR, Walters E, Illich P, Weeks J, Lewin M. 2001 Defensive responses of larval Manduca sexta and their sensitization by noxious stimuli in the laboratory and field. J. Exp. Biol. **204**, 457–469. (10.1242/jeb.204.3.457) 11171298

[7] Kondakath G, Trimmer BA. 2024 Characterization of a rapid avoidance behavior in Manduca sexta larvae in response to noxious stimuli. J. Exp. Biol. **227**, jeb248012. (10.1242/jeb.248012) 39475103 PMC11698039

[8] Weeks JC, Jacobs GA. 1987 A reflex behavior mediated by monosynaptic connections between hair afferents and motoneurons in the larval tobacco hornworm, Manduca sexta. J. Comp. Physiol. **160**, 315–329. (10.1007/BF00613021) 3572850

[9] McMackin MZ, Lewin MR, Tabuena DR, Arreola FE, Moffatt C, Fuse M. 2016 Use of von Frey filaments to assess nociceptive sensitization in the hornworm, Manduca sexta. J. Neurosci. Methods **257**, 139–146. (10.1016/j.jneumeth.2015.09.015) 26432932 PMC4662919

[10] Babcock DT, Landry C, Galko MJ. 2009 Cytokine signaling mediates UV-induced nociceptive sensitization in Drosophila larvae. Curr. Biol. **19**, 799–806. (10.1016/j.cub.2009.03.062) 19375319 PMC4017352

[11] Im SH, Galko MJ. 2012 Pokes, sunburn, and hot sauce: Drosophila as an emerging model for the biology of nociception. Dev. Dyn. **241**, 16–26. (10.1002/dvdy.22737) 21932321 PMC3258975

[12] Im SH, Takle K, Jo J, Babcock DT, Ma Z, Xiang Y, Galko MJ. 2015 Tachykinin acts upstream of autocrine Hedgehog signaling during nociceptive sensitization in Drosophila. eLife **4**, e10735. (10.7554/eLife.10735) 26575288 PMC4739760

[13] Im SH, Patel AA, Cox DN, Galko MJ. 2018 Drosophila insulin receptor regulates the persistence of injury-induced nociceptive sensitization. Dis. Model. Mech. **11**, dmm034231. (10.1242/dmm.034231) 29752280 PMC5992604

[14] Tabuena DR, Solis A, Geraldi K, Moffatt CA, Fuse M. 2017 Central neural alterations predominate in an insect model of nociceptive sensitization. J. Comp. Neurol. **525**, 1176–1191. (10.1002/cne.24124) 27650422 PMC5258852

[15] Mukherjee R, Trimmer BA. 2020 Local and generalized sensitization of thermally evoked defensive behavior in caterpillars. J. Comp. Neurol. **528**, 805–815. (10.1002/cne.24797) 31644815

[16] MacWilliam D, Arensburger P, Higa J, Cui X, Adams ME. 2015 Behavioral and genomic characterization of molt-sleep in the tobacco hornworm, Manduca sexta. Insect Biochem. Mol. Biol. **62**, 154–167. (10.1016/j.ibmb.2015.01.012) 25661727

[17] Adamo SA, Kovalko I, Turnbull KF, Easy RH, Miles CI. 2016 The parasitic wasp Cotesia congregata uses multiple mechanisms to control host (Manduca sexta) behaviour. J. Exp. Biol. **219**, 3750–3758. (10.1242/jeb.145300) 27634401

[18] Ache JM, Namiki S, Lee A, Branson K, Card GM. 2019 State-dependent decoupling of sensory and motor circuits underlies behavioral flexibility in Drosophila. Nat. Neurosci. **22**, 1132–1139. (10.1038/s41593-019-0413-4) 31182867 PMC7444277

[19] Mongeluzi DL, Hoppe TA, Frost WN. 1998 Prepulse inhibition of the Tritonia escape swim. J. Neurosci. **18**, 8467–8472. (10.1523/JNEUROSCI.18-20-08467.1998) 9763489 PMC6792843

[20] Frost WN, Tian LM, Hoppe TA, Mongeluzi DL, Wang J. 2003 A cellular mechanism for prepulse inhibition. Neuron **40**, 991–1001. (10.1016/s0896-6273(03)00731-1) 14659097

[21] Sandstrom DJ, Weeks JC. 1996 Novel dual innervation of a larval proleg muscle by two similar motoneurons in the tobacco hornworm Manduca sexta. J. Exp. Biol. **199**, 775–791. (10.1242/jeb.199.4.775) 8788086

[22] Trimmer BA, Weeks JC. 1991 Activity-dependent induction of facilitation, depression, and post-tetanic potentiation at an insect central synapse. J. Comp. Physiol. A **168**, 27–43. (10.1007/BF00217101) 2033567

[23] Cattaert D, El Manira A, Bévengut M. 1999 Presynaptic inhibition and antidromic discharges in crayfish primary afferents. J. Physiol. Paris **93**, 349–358. (10.1016/s0928-4257(00)80062-5) 10574123

[24] Kuffler SW, Eyzaguirre C. 1955 Synaptic inhibition in an isolated nerve cell. J. Gen. Physiol. **39**, 155–184. (10.1085/jgp.39.1.155) 13252239 PMC2147519

[25] Dominick OS, Truman JW. 1986 The physiology of wandering behaviour in Manduca sexta: III. Organization of wandering behaviour in the larval nervous system. J. Exp. Biol. **121**, 115–132. (10.1242/jeb.121.1.115) 3958674

[26] Kent KS, Hildebrand JG, Wiesel TN. 1987 Cephalic sensory pathways in the central nervous system of larval Manduca sexta (Lepidoptera: Sphingidae). Phil. Trans. R. Soc. Lond. B **315**, 1–36. (10.1098/rstb.1987.0001) 2881311

[27] Pfeiffer K, Homberg U. 2014 Organization and functional roles of the central complex in the insect brain. Annu. Rev. Entomol. **59**, 165–184. (10.1146/annurev-ento-011613-162031) 24160424

[28] Gibbons M, Sarlak S, Chittka L. 2022 Descending control of nociception in insects? Proc. R. Soc. B **289**, 20220599. (10.1098/rspb.2022.0599) PMC9257290 35858073

[29] Bachtel ND, Hovsepian GA, Nixon DF, Eleftherianos I. 2018 Allatostatin C modulates nociception and immunity in Drosophila. Sci. Rep. **8**, 7501. (10.1038/s41598-018-25855-1) 29760446 PMC5951828

[30] Hu C *et al*. 2017 Sensory integration and neuromodulatory feedback facilitate Drosophila mechanonociceptive behavior. Nat. Neurosci. **20**, 1085–1095. (10.1038/nn.4580) 28604684 PMC5931224

[31] Hu Y, Wang C, Yang L, Pan G, Liu H, Yu G, Ye B. 2020 A neural basis for categorizing sensory stimuli to enhance decision accuracy. Curr. Biol. **30**, 4896–4909. (10.1016/j.cub.2020.09.045) 33065003 PMC7755697

[32] Gibbons M, Versace E, Crump A, Baran B, Chittka L. 2022 Motivational trade-offs and modulation of nociception in bumblebees. Proc. Natl Acad. Sci. USA **119**, e2205821119. (10.1073/pnas.2205821119) 35881793 PMC9351458

[33] Ohashi H, Sakai T. 2018 Leucokinin signaling regulates hunger-driven reduction of behavioral responses to noxious heat in Drosophila. Biochem. Biophys. Res. Commun. **499**, 221–226. (10.1016/j.bbrc.2018.03.132) 29559237

[34] Chen Y, Veenstra JA, Hagedorn H, Davis NT. 1994 Leucokinin and diuretic hormone immunoreactivity of neurons in the tobacco hornworm, Manduca sexta, and co-localization of this immunoreactivity in lateral neurosecretory cells of abdominal ganglia. Cell Tissue Res. **278**, 493–507. (10.1007/BF00331367) 7850860

[35] Nakamizo-Dojo M, Ishii K, Yoshino J, Tsuji M, Emoto K. 2023 Descending GABAergic pathway links brain sugar-sensing to peripheral nociceptive gating in Drosophila. Nat. Commun. **14**, 6515. (10.1038/s41467-023-42202-9) 37845214 PMC10579361

[36] Hossie TJ, Sherratt TN. 2013 Defensive posture and eyespots deter avian predators from attacking caterpillar models. Anim. Behav. **86**, 383–389. (10.1016/j.anbehav.2013.05.029)

[37] Hossie TJ, Sherratt TN. 2014 Does defensive posture increase mimetic fidelity of caterpillars with eyespots to their putative snake models. Curr. Zool. **60**, 76–89. (10.1093/czoolo/60.1.76)

[38] Nishino H. 2004 Motor output characterizing thanatosis in the cricket Gryllus bimaculatus. J. Exp. Biol. **207**, 3899–3915. (10.1242/jeb.01220) 15472021

[39] Pflüger HJ, Büschges A, Bässler U. 2021 Historical review on thanatosis with special reference to the work of Fritz Steiniger. In Death-feigning in insects: mechanism and function of tonic immobility (ed. M Sakai), pp. 15–21. Singapore: Springer. (10.1007/978-981-33-6598-8_2)

[40] Bell RA, Joachim FG. 1976 Techniques for rearing laboratory colonies of tobacco hornworms and pink bollworms. Ann. Entomol. Soc. Am. **69**, 365–373. (10.1093/aesa/69.2.365)

[41] Belanger JH, Bender KJ, Trimmer BA. 2000 Context dependency of a limb withdrawal reflex in the caterpillar Manduca sexta. J. Comp. Physiol. A **186**, 1041–1048. (10.1007/s003590000161) 11195280

[42] Caron DP, Rimniceanu M, Scibelli AE, Trimmer BA. 2020 Nociceptive neurons respond to multimodal stimuli in Manduca sexta. J. Exp. Biol **223**, jeb218859. (10.1242/jeb.218859) 31932302

[43] R Core Team. 2020 R: a language and environment for statistical computing. Vienna, Austria: R Foundation for Statistical Computing. See http://www.R-project.org/.

[44] Wickham H. 2016 Ggplot2: elegant graphics for data analysis, 2nd edn. Cham, Switzerland: Springer Nature. (10.1007/978-3-319-24277-4)

[45] Bates D, Mächler M, Bolker B, Walker S. 2015 Fitting linear mixed-effects models using lme4. J. Stat. Softw. **67**, 1–48. (10.18637/jss.v067.i01)

[46] Lenth RV *et al*. 2023 emmeans: Estimated Marginal Means, aka Least-Squares Means. See https://CRAN.R-project.org/package=emmeans.

[47] Kondakath G, Messinger IM, Veliko-Shapko A, Trimmer BA. 2025 Data from: Caterpillars suppress nocifensive behaviours during the quiescent ‘sphinx’ state. Figshare. https://figshare.com/s/fe9eda8973e68b21cb9910.1098/rsbl.2025.012440829651

[48] Kondakath G, Messinger IM, Veliko-Shapko A, Trimmer B. 2025 Supplementary material from: Caterpillars suppress nocifensive behaviours during the quiescent ‘sphinx’ state. Figshare. (10.6084/m9.figshare.c.7951324)40829651

